# Efforts to Ban the Routine Tail Docking of Pigs and to Give Pigs Enrichment Materials via EU Law: Where Do We Stand a Quarter of a Century on?

**DOI:** 10.3390/ani9040132

**Published:** 2019-03-29

**Authors:** Elena Nalon, Nancy De Briyne

**Affiliations:** 1 Eurogroup for Animals, Rue Ducale 29, B-1000 Brussels, Belgium; 2 Federation of Veterinarians of Europe, Avenue Tervueren 12, 1040 Brussels, Belgium

**Keywords:** animal welfare, EU policy, pig directive, enrichment materials, mutilations, straw, swine, tail biting, veterinarian

## Abstract

**Simple Summary:**

Enforcing legislation on the welfare of pigs is currently one of the European Commission’s priorities in the area of animal welfare. This article focuses on the legal ban on the routine docking of tails and the provision of enrichment to pigs within the European Union. It provides a chronological overview of the steps that have been taken by European policy makers to promote the correct implementation and enforcement of the law. In addition, it analyses the current state of play, and presents a reflection on possible future scenarios.

**Abstract:**

In its role as guardian of the Treaties, the European Commission must ensure that Member States enforce EU law within their territories. If adequate enforcement is found to be wanting, the Commission also has the power to take infringement procedures as a corrective measure. The case of Directive 120/2008/EC on the protection of pigs is problematic, as only a few Member States are respecting the ban on routine tail docking, whilst not all pigs are given (adequate) enrichment materials. Twenty-five years after the first EU-wide legal ban on routine tail docking came into force, we are faced with an unprecedented situation that may lead to infringement procedures against more than 20 Member States. This paper describes the various steps that led to the development of the EU law designed specifically to safeguard the welfare of pigs. It lists the numerous efforts (research studies, study visits, recommendations, audits, reports, factsheets, action plans, etc.), undertaken by European decision makers to assist Member States in their efforts to better implement and enforce the relevant rules. Finally, the paper further analyses the current state of play and presents a reflection on possible future scenarios.

## 1. Introduction

Pigs are kept for meat in most European Union (EU) countries. In total in 2017, around 149 million pigs were kept in the EU, with the largest numbers in Spain (31 million), Germany (26 million), France (14 million), Denmark (13 million), the Netherlands (12 million) and Poland (11 million) [1]. Pigs in the EU are mainly kept indoors, with smaller farms having been replaced in the last decade by medium to large-scale farms [2]. Tail biting is a damaging behavior derived from an interplay of factors, some relating to the individual animal concerned (e.g., sex, genetics), others dependent upon how the animals are managed (e.g., lack of manipulable materials, poor climate, feeding problems, dysfunctional social structure, and poor pen layout [3]). 

Tail biting is not only an animal welfare problem, as bitten pigs suffer from pain, as well as stress, and can develop infections, but it also carries an economic cost, as such infections can cause carcass condemnations at slaughter [3,4]. However, tail docking per se does not prevent tail biting, and there is evidence that tail biting can be prevented or mitigated in undocked pigs by improving management and housing [3,5]. 

Under most commercial rearing conditions, the presence of exploration and foraging opportunities is typically minimal or absent. The absence of foraging substrates has been identified as one major, albeit not isolated, risk factor for tail biting [6]. The importance of providing enrichment materials to pigs and reducing stocking densities to manage the risk of tail biting was already stressed in a 1997 report by the Scientific Veterinary Committee, where it stated that *“tail-biting can largely be prevented by providing straw or other manipulable materials and keeping pigs at a stocking density which is not too high.”* [7]. In effect, the availability of enrichment materials, especially straw, in animal production systems is widely presumed to be beneficial for the health and welfare of the animals [8]. Pigs in particular have an intrinsic need to explore, even when enough feed is provided. Under semi-natural conditions, domestic pigs have been observed to spend up to 6–8 h foraging for food [9,10]. Foraging activities include rooting, but also grazing and browsing [9]. Exploration of the surroundings is also innate behavior for pigs, occurring even in the absence of external stimuli [10,11]. Appropriate enrichment materials provide pigs with the opportunity to express foraging, rooting, and chewing behaviors, and reduce the occurrence of oral stereotypies [8]. They can also stimulate and satisfy play behavior, which is a very important social aspect to gain social skills, especially in young pigs. Specific edible materials such as straw can supplement fiber to the diet [12] and, if used as bedding, can increase thermal comfort in colder climates. Conversely, it is acknowledged that a lack of environmental enrichment is among the factors determining harmful consequences such as tail biting, ear biting, or aggressive behavior [13,14]. 

**Figure 1:**
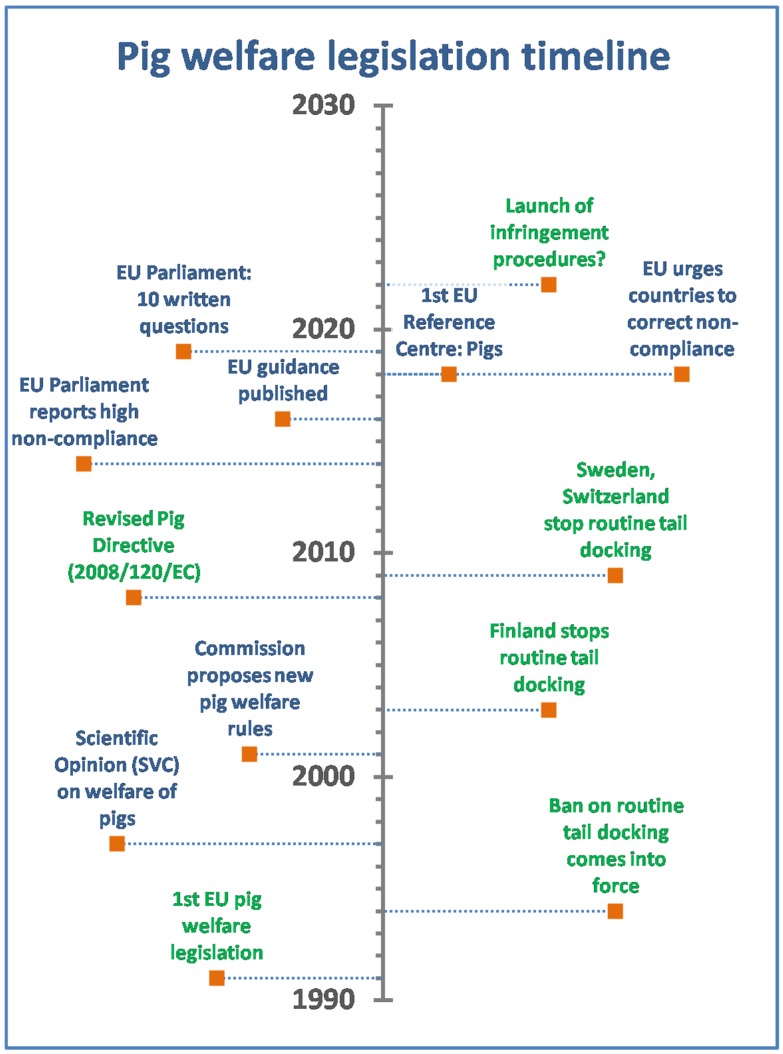
Schematic timeline of EU pig welfare legislation (in green) and non-legislative initiatives (in blue) on the topic of pig welfare with special reference to the ban on routine tail docking.

The first EU-wide rules on pig welfare were established in 1991 via Directive 91/630/EEC [15]. This Directive was subsequently amended several times and was substantially updated by Directive 120/2008/EC (henceforth, the Pig Directive [16]). The prohibition on routine tail docking has been in place since 1994, along with the clear stipulation that, to prevent tail biting, enrichment materials such as straw or other suitable materials should be provided to satisfy the behavioral needs of pigs. The Pig Directive specifies the measures that must be undertaken before a farmer can resort to tail docking (i.e., addressing management, stocking density, and providing specific enrichment materials). Nevertheless, tail docking is still practiced routinely in many EU countries [4,17], in violation of these provisions. In Finland and Sweden, due to stricter national rules compared to EU legislation, tail docking is no longer allowed. Outside the EU, in Norway and Switzerland, less than 5% of pigs are tail-docked [4,17]. However, routine tail docking is also carried out in many other countries beyond the Union’s borders. Although the current EU pig welfare legislation is in need of updating, as science and technology are constantly evolving, both the enforcement of the provision relating to enrichment and the ban on routine tail docking of pigs have proven to be extremely problematic. In this paper we will examine the EU regulatory background, describe the current situation, and delineate the possible future perspectives on these specific aspects. For an overview at a glance of legislative and non-legislative initiatives leading to the current state of play, see Figure 1.

## 2. The Process Leading to Current EU Pig Welfare Legislation 

### 2.1. First European Pig Welfare Legislation: 1994 

The first EU pig welfare legislation [15] was agreed in 1991. At that time, 12 countries (Belgium, Denmark, France, Germany, Greece, Ireland, Italy, Luxemburg, Netherlands, Portugal, Spain and the UK) were part of the then European Community. The drafting of European animal welfare legislation was initiated as a result of the industrialization of the livestock sector, and the evidence of poor welfare on some farms [18]. 

It mandated that “*no tail docking must be carried out routinely but only when there is evidence that injuries to other pigs’ tails have occurred as a result of not tail docking’* and ‘*that in addition to the measures normally taken to prevent tail-biting and in order to satisfy their behavioural* (sic) *needs, all pigs, taking into account environment and stocking density, must be able to obtain straw or any other suitable material or objects*”. All member countries had to transpose this Directive into their national legislation, preparing regulations and administrative provisions, including sanctions, by 1 January 1994.

### 2.2. 1997–2001 Years of Reflection 

Article 6 of the 1991 Directive required the Commission to submit a report by 1 October 1997 on intensive pig-rearing systems that complied with the welfare requirements of pigs from the pathological, zootechnical, physiological and behavioral points of view, and on the socio-economic implications of the different systems. The report was delivered by a Scientific Veterinary Committee [7] and provided a detailed analysis of the knowledge (at that time) on the physiology and behavior of domestic pigs and on the health and welfare of intensively kept pigs. More specifically, the report gave 88 recommendations on how the welfare of pigs could be improved, while also taking into account the socio-economic implications. It described in detail the problems seen with tail biting, and a lack of enrichment materials. It confirmed that tail biting should be solved by improving management, rather than tail docking, and stressed the importance of providing enrichment materials to all pigs, and decreasing stocking densities. Four years later, in 2001, the European Commission presented a proposal to the co-legislators, the Council and the European Parliament, stating the intention to revise the 1991 Directive [19,20]. The European Economic and Social Committee (EESC) also presented its opinion on the proposal. Notably the EESC regretted that although the Opinion of the Veterinary Scientific Committee had advised on the minimum space requirements for pigs, the Commission had decided to wait to introduce such rules until the sector was in better financial health. The EESC argued that insufficient space for animals leads to tail biting, meaning that tail docking would remain necessary [21].

### 2.3. Amended European Pig Directive: 2008

Some aspects of the 1991 Directive were periodically amended, albeit in a piecemeal manner, until the Council of the European Union adopted a substantial revision in 2008. Council Directive 2008/120/EC [16] applied from March 2009, by then covering 27 Member States. Among other important provisions, the Pig Directive reiterated and emphasized the earlier ban on routine tail docking, and the obligation to provide enrichment materials, specifying this time that there should be “*permanent access* to a *sufficient quantity”* of material to enable proper investigation and manipulation activities. For the first time, the Directive also included a list of materials that can be considered adequate enrichment, namely *“straw, hay, wood, sawdust, mushroom compost, peat or a mixture of such, which does not compromise the health of the animals”* (Annex I, Chapter 1, point 4).

## 3. 2008–2019: Implementation and (Lack of) Enforcement of Certain Provisions of the Pig Directive

All Member States transposed the Pig Directive into their national law before the legal deadline. Notably in Finland, tail docking had already been banned from 2003, whereas Sweden introduced a total ban through transposing the Directive. These are hitherto the only EU Member States in which demonstrably pig tails are routinely kept intact. In the remainder, it very quickly became clear that the majority (or a large proportion) of pigs continued to be routinely docked, and were not habitually provided with suitable and sufficient enrichment materials. 

**Figure 2:**
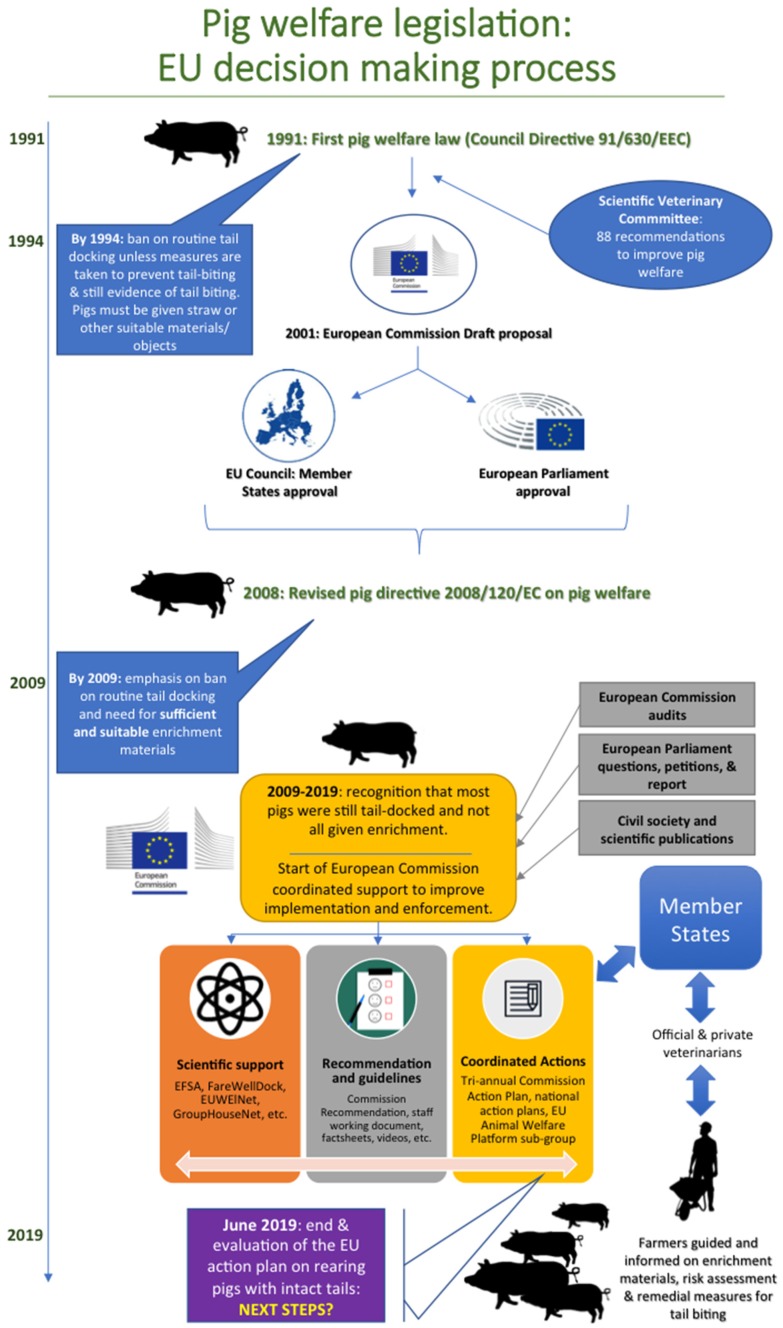
Schematic representation of the temporal sequence of EU-level decision-making processes leading to current pig welfare legislation and actions undertaken by the European Commission to increase Member State compliance with the Pig Directive.

The widespread failure, not only to enforce the ban on routine tail docking, but also to ensure provision of sufficient and suitable enrichment materials, was brought to the attention of the European Institutions in several ways in the years that followed (for a schematic overview, see Figure 2). 

The seventh European Parliament closely scrutinized the lack of implementation of the Pig Directive, with its Members submitting 12 written parliamentary questions to the Commission between 2009 and 2014. A further 10 have been submitted by Members of the eighth European Parliament at the time of writing [22]. In addition, the European Parliament Petitions Committee, a special committee to which all European citizens or residents have the right to send a petition [23] on matters of Union concern, received three petitions on the issue of tail docking and enrichment materials. At the time of writing, these petitions are still open. As a result, in 2014 the Policy Department of the European Parliament prepared a comparative study entitled “In–depth analysis on the routine tail docking of pigs” based on data from Denmark, Sweden, the United Kingdom, Germany, Netherlands and Belgium [24]. This study revealed a *“persisting high rate of non-compliance in the large majority of Member States”* with the EU ban on routine tail docking. It recommended that the European Commission be bolder and adopt *“a stricter enforcement policy”* which, alongside non-legislative tools such as guidelines and e-learning tools for farmers, should also include infringement procedures, considering that *“the mere prospect of serious action may prompt Member States to comply”*. In addition, the European Parliament’s Intergroup for the Welfare and Conservation of Animals [25], a cross-group forum open to all Members of the European Parliament with an interest in Animal Welfare, sent multiple letters to the responsible European Commissioners (in both Parliamentary terms), asking them to undertake urgent measures to address the lack of compliance. Subsequent meetings were organized with the respective Commissioners to discuss the way forward, and to request the instigation of infringement procedures.

Serious concerns about the lack of enforcement of the Pig Directive have also been forthcoming from civil society: In 2018, an EU-wide public-facing campaign, coordinated by Eurogroup for Animals [26], collected over one million signatures from European citizens, calling on the European Commission and Member States to fully enforce this law, and to take actions to phase-out the routine surgical castration of piglets without pain relief (for an overview of this issue, see [27]). 

Finally, in 2018, the European Court of Auditors issued a report [28] examining the effectiveness of the European Commission’s spending on animal welfare measures. The results concerning the Pig Directive are unambiguous: “…*weaknesses still persisted in some areas related to welfare issues on the farm (in particular, the routine tail docking of pigs”* (p. 5). Additionally, the Court of Auditors found that in two Member States *“Pigs in farms receiving measure 14 support* (i.e., rural development funds for animal welfare under the Common Agricultural Policy) *had their tails docked and did not have access to sufficient enrichment material, as required by the legislation”.* This indicates that some farms that are routinely tail-docking pigs are nonetheless receiving public funds for the purposes of improving animal welfare (measure 14). 

The Commission also allocates resources to checking the enforcement of EU legislation. The Directorate General For Health and Food Safety has a specific auditing department (formerly the Food and Veterinary Office, currently Health, Food Audits and Analysis), that verifies that EU legislation on animal health, animal welfare and food safety is properly implemented and enforced [29]. Audits from this unit have confirmed widespread problems with the implementation and enforcement of the ban on routine tail docking, in addition to serious shortcomings concerning the provision of adequate enrichment [30]. To address the situation, an overview report [31] was published in 2016, based on study visits to Finland, Sweden and Switzerland, showcasing good practices for the production of pigs with intact tails, and highlighting why this is not widely practiced in the EU (economics, tail biting and slurry handling in relation to enrichment materials). Nonetheless, the results of the most recent official audits carried out in Germany, the Netherlands, Italy, Spain, and Denmark—the main European pig producing countries—between 2016 and 2018, demonstrate the ubiquitous nature of the problem: 95 to 100% of pigs are still being tail docked [30]. 

## 4. Corrective Instruments Available to the European Commission

Member States have the responsibility to implement and enforce EU legislation (directives, regulations, and other legally binding acts). For its part, the European Commission, in its role as the “guardian of the Treaties” and as the Union’s executive, has a duty to exert vigilance and ensure proper and timely implementation and enforcement of EU legislation [32]. If one or more Member States fail to transpose and/or enforce EU legislative provisions, the Commission can use various instruments to correct the situation. Such instruments can be broadly classified into two categories: The enforcement approach (coercive) and the management approach (non-coercive), where the Commission has a tradition of associating the two in order to hasten Member State compliance with EU law [32]. 

The enforcement approach (infringement procedures) is based on the experience that financial sanctions—either threatened or enacted—can deter Member States from ignoring their obligations to enforce Union law, as this becomes damaging both in economic and reputational terms to the Member State concerned [32]. Infringement procedures have three stages: formal notice, reasoned opinion and referral to the Court of Justice (CoJ). The first stage is a *letter of formal notice* by the Commission, requesting information to one or more Member States. If—after receiving the requested information—the Commission finds that the Member State is failing to fulfill its obligations under EU law, it will send a formal request to comply, explaining the reasons why it considers that the country in question is breaching EU law. This second stage is called *reasoned opinion*. The Member State is required to inform the Commission of measures taken to comply within two months. If non-compliance persists, the Commission can initiate the third stage and refer the matter to the Court of Justice (CoJ), and may also ask the CoJ to impose financial penalties (*sanctions*) to the Member State. This stage is called the *Referral to the CoJ*. If the CoJ finds that the Member State has breached EU law, the national competent authorities must take corrective measures. 

Financial penalties can also be imposed by the CoJ on the request of the European Commission if, after a first CoJ judgment, a member state still fails to comply with EU legislation. In this case the member state is referred to the CoJ for a second time [33]. Most infringement procedures do not reach the stage of referral to the CoJ, but are resolved earlier, as this system of public “escalation of pressure” is very effective in creating negative publicity for the Member State(s) concerned, and there is a common interest in avoiding costly litigation procedures [33]. Examples of (now closed) infringement procedures started by the Commission in the field of animal welfare include those against Italy for failure to first transpose (Infringement n. 20044171, 2005–2006), and then enforce (Infringement n. 20112231, 2012–2015), the Laying Hen Directive concerning the ban on barren “battery” cages, and that against Greece for failure to enforce the same Directive (Infringement n. 20112230, 2012–2015). Additionally, in February 2013 the Commission sent letters of formal notice to nine Member States (Belgium, Cyprus, Denmark, France, Germany, Greece, Ireland, Poland and Portugal) for failure to address deficiencies in the implementation of the group housing of pregnant sows, which had come into force in January 2013 [34]. 

The management approach is based on the assumption that Member States must be supported (economically, and with information and guidance) in the adaptations needed to enforce EU legislation [32]. According to some political scientists, the strategy of associating punishment (or the threat of punishment) with persuasive methods has historically proven to be more effective than employing only coercive methods. 

## 5. 2012–2016: A Management-Based Approach to Improve Enforcement of the Pig Directive

In 2016, having noted the unsatisfactory implementation and enforcement of the Pig Directive, the Commission set about remedying this situation, opting for a management-based approach. This approach was carried out in stages, with a period of preparatory actions followed by an operational phase that is still ongoing at the time of writing.

### 5.1. Scientific Preparatory Work 

To increase compliance with the provisions of the Pig Directive and fill knowledge gaps, the Commission funded a series of preparatory studies carried out by scientific consortia (e.g., EUWelNet [35], FareWellDock [36], GroupHouseNet [37]). The studies were aimed at investigating and clarifying practical aspects linked to the management of pigs with intact tails. These projects produced detailed results concerning the effects of different types of enrichment on tail biting, while also taking into account the role of other important factors, such as ventilation, feeding, management, the reduction of stocking densities, and herd health status.

In 2014, the Panel on Animal Health and Welfare (AHAW) of the European Food Safety Authority (EFSA) updated a previous scientific opinion on risk factors for tail biting and possible means to reduce tail docking [17], with new information specifically on manipulable materials and animal-based indicators for pig welfare [6]. The updated scientific review produced guidance on how to practically assess the effectiveness of manipulable materials provided to pigs, as well as the presence and relative importance of risk factors for tail biting. In its conclusions, the EFSA panel found that the presence of manipulable materials is important at all stages of life for the pigs, in order to minimize the risk of undesirable behaviors, including tail biting. The final report stressed the importance of identifying problems and adapting management and environmental factors to control the risk of tail biting. Finally, it recommended two “risk assessment toolboxes” for on-farm use—one to assess the risk of tail biting, and another to assess the adequacy of the enrichment materials provided—that included a combination of resource-based and animal-based indicators. 

### 5.2. Identifying Best Practices and Giving Guidance: The Commission Recommendation and Staff Working Document

On the basis of those preparatory studies, the European Commission set up an expert working group to develop official guidance. The guidance, produced after interservice consultation (consultation within all affected directorate generals of the European Commission), was adopted in 2016. It consisted of two separate documents, the Commission Recommendation 2016/336 [38] “on the application of Council Directive 2008/120/EC laying down minimum standards for the protection of pigs as regards measures to reduce the need for tail-docking”, and a Staff Working Document [39] “on best practices with a view to the prevention of routine tail-docking and the provision of enrichment materials to pigs”. 

The legally binding Commission Recommendation 2016/336 [38] tasks Member States with ensuring that farmers carry out a risk assessment for factors potentially leading to tail biting, and enact corrective measures for each recorded risk factor. It also importantly specifies that enrichment materials must be *“edible, chewable, investigable and manipulable”*, (and explains what these terms mean). Additionally, these materials should be “*of sustainable interest*”, meaning that they should be able to stimulate the exploratory behavior of pigs, and that they should be regularly replaced and replenished. The Recommendation further classifies the conditions that enrichment materials must satisfy to comply with the legal requirements of the Pig Directive. The various admissible materials are ranked based on their adequacy and interest for the animals: optimal materials can be used on their own (examples: straw, fodder), suboptimal materials (examples: wood blocks, ropes) must be used in combination with other materials, whereas materials of marginal interest (examples: plastic balls, metal chains) must be used in combination with optimal or suboptimal materials. The Recommendations also advise farmers and competent authorities to use a combination of resource- and animal-based indicators to assess whether the materials provided are adequate. 

The Staff Working Document [39] is meant to give guidance to the pig sector and help competent authorities of Member States understand the rationale behind the Recommendation, while explaining the main scientific principles of pig health and welfare. It provides detailed indications on the types of materials that can be used as enrichment, and on other managerial factors that can help prevent or reduce tail biting (diet, health, thermal comfort, air quality, etc.). The document also provides advice on how to address bouts of tail biting in a herd or group of pigs.

### 5.3. The 2017–2019 EU Action Plan on Rearing Pigs with Intact Tails

In 2017 the Commission launched a tri-annual EU action plan (2017–2019) based on guidance, the exchange of best practices, study visits, educational materials, stakeholder meetings and consultations, as the main tool to improve compliance with the Pig Directive. The Commission also requested all Member States to provide by December 2018 national action plans detailing how they intend to reach compliance with the requirements of the Directive, with a specific stress on the avoidance of routine tail docking. In 2017, in order to assist Member States, The Directorate-General for Health and Food Safety (DG SANTÉ) produced a series of factsheets [5] covering aspects of pig health and welfare that can contribute to eliminating the need for routine tail docking. The factsheets are accompanied by two videos showing the best practices for rearing pigs with intact tails under completely different systems: the first video shows an example from a big industrial farm in Finland (3000 breeding sows; [40]); the second is about an important Italian producer rearing heavy pigs for the Parma Ham consortium [41]. As pig production systems across the EU vary depending on the geographical region, the library may be expanded to include more examples of best practice from different countries (European Commission, personal communication).

### 5.4. The European Union Platform on Animal Welfare

In January 2017, following requests from several Member States, the European Commission created the EU Platform on Animal Welfare [42], in order to provide EU and national level agencies with a forum to interact with industry, civil society and academia. The Platform is a Commission expert group that will run until 31 December 2019, and at the time of writing it is not known whether it will be renewed. It brings together 75 institutional, industrial and non-governmental stakeholders, including representatives of all Member States. It has among its main goals to assist the Commission with the development and exchange of coordinated actions to improve enforcement of current animal welfare legislation, which is problematic in several areas, and to this aim it has created several thematic sub-groups. One such sub-group was created in September 2018 to work on pig welfare issues [43]. The objective of the pig sub-group is to advise on how the risk of tail biting can be reduced by meeting the relevant legal requirements in the Pig Directive. Among other activities, the group will also work on animal-based indicators that competent authorities can use to assess the health and welfare status of pigs on farms during official inspections, and which can also be used by quality assurance and agri-food schemes. The first deliverables of the pig sub-group are expected by the end of 2019. 

### 5.5. The first EU Reference Centre on Animal Welfare Dedicated to Pig Welfare

On 5 March 2018 the European Commission designated a first European Union Reference Centre for Animal Welfare [44]. This first Centre, a consortium of three research institutes, is dedicated to pig welfare. Its designation will be reviewed every five years. Its task is to provide technical support and coordinated assistance to the Member States in carrying out official controls in the field of pig welfare and to disseminate good practices. The Centre will also provide scientific and technical expertise, carrying out studies and developing methods for assessing and improving pig welfare. The Centre recently published its first work plan [45] in consultation with EU Member States and other stakeholders and is collaborating with the EU Animal Welfare Platform to communicate results and inform stakeholders on relevant developments. 

## 6. The Future: What Can/Will Happen Next? 

2019 will be a year of change within key EU Institutions, not only due to the high probability of the withdrawal of the United Kingdom from the Union, but also as a new European Parliament will be elected, and a new European Commission will be inaugurated. In July 2019, the EU action plan to facilitate the rearing of pigs with intact tails will also end; it will be evaluated and a future approach will need to be decided. The installation of a new executive from the autumn of 2019 will also potentially mean a reshuffling of priorities, and therefore the future approach towards enforcement is currently uncertain. In our opinion, the following scenarios are possible:

1/The new Commission decides to prolong the action plan and continue with a guidance-based approach, still postponing infringement procedures.
2/The new Commission decides to use coercive methods (infringement procedures) with or without a continuation of guidance of Member States to obtain better enforcement.

It must be considered that enforcing EU law is not only a prerogative, but an obligation of the European Commission. It therefore seems implausible that efforts to enforce pig welfare provisions will be disregarded. 

## 7. Discussion

The first ban on routine tail docking across the EU dates from 1994. This means that 1 January 2019 marked the 25th birthday of this provision, which is still largely unenforced by Member States. Whilst infringement procedures may be on the horizon, it is equally plausible that the Commission will only continue to offer guidance and assistance to Member States in their efforts to improve compliance. 

Under the current circumstances, the Commission would have to launch over 20 infringement procedures (considering the majority of Member States are non-compliant). However, this is not an exceptional circumstance. By way of an example, as regards air pollution (Directive 2008/50), the Commission declared in 2013 that it had started infringement procedures against 17 Member States for non-compliance with maximum legal limits for PM10 [46]. Until now, the Court of Justice has issued more than 30 judgments against Member States for not complying with the Directive on urban waste water (Directive 91/271), and an equal number with regard to the Directive on nitrates from agricultural production (Directive 91/676). When requested by the Commission, Court of Justice judgments can include financial sanctions that, within the expected spending capacities of the Member State(s), are meant to be conducive to corrective actions (e.g., up to tens of million euros per semester of persistent non-compliance, plus a compensatory bulk sum, and the legal costs). Additionally, the Commission could at least dissuade Member States from some of the worst practices by introducing stricter criteria for obtaining funding under the program for the Promotion of Agricultural Products (part of the budget of the Common Agricultural Policy) managed by the Consumer, Health, Agriculture and Food Executive Agency (CHAFEA). Each year the Commission establishes a line of funding for consortia of European producers to promote “quality products” (products with geographical indication, organic products, etc.) on export markets, and sometimes on the internal market [47]. In recent years, consortia of pig producers located in Member States that—based on the official audit reports by DG SANTÉ—do not respect the Pig Directive regarding the provision of adequate enrichments and the ban on routine tail docking, have been given several million euros in co-funding to promote their pork products abroad. The Commission could, therefore, decide only to give funding to European producers complying completely with the Pig Directive. 

What factors have led the European Commission to use a management-based approach over coercive methods? One possible reason is that the EU pig sector is currently faced with the saturation of the demand for pig meat on the internal market, coupled with an animal health crisis due to African Swine Fever (ASF). ASF is a highly contagious viral disease that affects wild boars and domestic pigs, with a high degree of morbidity and mortality, and which disrupts exports and leads to substantial economic losses [48]. It should be noted that the EU has a self-sufficiency of about 111% for pig meat, and exports about 13% of its total production. The EU is the first global exporter of pig meat worldwide [49] and arguably wants to maintain this position.

The European livestock sector is concerned that EU animal welfare legislation is causing a competitive disadvantage for EU products on the global market [50,51]. In 2018, to respond to these concerns, the Commission published a ‘Report on the impact of animal welfare international activities on the competitiveness of European livestock producers in a globalized world’ [52]. The report concluded that the EU has played a prominent and decisive role in raising global awareness on animal welfare, and that significant results have been achieved. The report also found that animal welfare standards have a limited impact on the competitiveness of EU producers on world markets: The overall costs of compliance with animal welfare standards remains very low compared to other production costs (such as the cost of labor and feed) that affect global competitiveness and influence world trade patterns. Therefore, EU animal welfare standards can even offer an opportunity to better valorize the added market value of EU products. However, specifically on the issue of stopping routine tail docking and providing enrichment materials, farmers are reluctant to implement changes. This can be explained through three main hypotheses: (i) due to the multifactorial nature of the problem, it may be difficult to predict and completely prevent episodes of tail biting; (ii) habit, i.e., the tendency to repeat habitual behavior, and limited knowledge about the alternatives; (iii) raising pigs with intact tails costs more, as a result of the need to decrease stocking density, provide more enrichment material, and the labor costs involved, including increased vigilance on the animals to identify (and remove, if necessary) those that have a tendency to bite [4]. The official audit reports of the Commission from Italy [53] and Spain [54] confirm this, and show that farmers consider it very difficult (if not impossible) to rear pigs with intact tails in the existing systems without giving pigs more space and more enrichment. They also consider that such improvements are not realistic under their countries’ commercial rearing conditions. 

On the other hand, farmers from countries with very different rearing systems are increasingly finding solutions that work for them. Successful examples of rearing pigs with intact tails have been reported from countries such as Germany, Denmark, Italy, and Ireland [55,56,57], as well as Finland and Sweden. Advantages reported by farmers include an increased pride in their work, an improvement in animal health with reduced need for antibiotics, and the possibility to obtain better prices on the market. The common denominator of these experiences is a substantial change of management, in which careful stockmanship, lower stocking densities, improved air and water quality, and the provision of a stress-free environment, play a major role. These factors are clearly summarized in the Commission’s online resources [5]. 

Member States are responsible for ensuring timely transposition and enforcement of EU law on their territories. There are various disincentives that can lead to lack of compliance, including financial costs, cost-benefit considerations, pressure from lobbies, and a lack of administrative infrastructure [32,58]. The official audits of the Commission stressed the lack of information in respect to the proportion of pigs that are tail docked, and the percentage of tail biting in different Member States. Measuring is a pre-condition for improvement. It is crucial to set up an effective monitoring program, both on the farm and at the abattoir, to record tail biting lesions and the percentage of docked pigs. These aspects will be addressed by the EU Reference Centre on Pig Welfare and within the dedicated sub-group of the EU Animal Welfare Platform. Developing reliable and harmonized animal-based indicators to measure animal welfare and assess compliance with legislative requirements has become a priority. Apart from drafting risk assessment templates, action plans to address identified risk factors, and taking controls seriously, Member States also have at their disposal a powerful instrument to help farmers financially to improve pig welfare, and eventually stop routine tail docking: Common Agricultural Policy spending under Pillar II. The rural development Regulation [59] provides Member States with possibilities to support investments specifically dedicated to animal welfare: so far these funds have been primarily used for the “modernization” of buildings, but incentives could also be given for reducing stocking densities, improving air and water quality, providing outdoor access where feasible, keeping animals on straw bedding where climate permits, or changing manure disposal systems so that straw can be provided as an enrichment. 

Last, but not least, the role of civil society in determining the speed of change cannot be underestimated. It is also thanks to the petitions initiated in the European Parliament by private citizens, the tenacious actions of Members of the European Parliament, the investigative materials, reports and signature collections received from non-governmental organizations, that the Commission has taken more decisive action. Indeed, external pressure (via the so-called “societal watchdogs”) is one of the fundamental instruments that the Commission uses to compensate for the lack of internal resources, when checking if EU legislation is being implemented and enforced [32]. 

To conclude, all the relevant actors—the Commission, the Member States, private and official veterinarians, farmers, and civil society at large—will have a role to play in increasing the chance of enforcing these 25-year old pig welfare provisions. The implications of keeping pigs with intact tails and providing them with meaningful and adequate enrichment are of course wider than a discussion about the purely technical solutions. Farm animal welfare is of paramount importance for European citizens, as shown by the results of the last special Eurobarometer on Animal Welfare [60], and scientific evidence is increasingly confirming the highly developed cognitive and emotional capabilities of farm animals, and the relevance of providing them with “lives worth living” [61]. Any long-standing and widespread lack of enforcement of EU legislation is thus detrimental not only for the animals, but also for the reputation of EU law as driver of better farm animal welfare globally [18]. A looming question remains unaddressed, and very difficult to answer. What if all efforts should fail? The consequences could potentially be profound, and call for a serious reflection on our current animal farming paradigm.
